# Design and Investigation of a Dynamic Auto-Adjusting Ejector for the MED-TVC Desalination System Driven by Solar Energy

**DOI:** 10.3390/e24121815

**Published:** 2022-12-13

**Authors:** Jianbo Ren, Heli Zhao, Min Wang, Chao Miao, Yingzhen Wu, Qiang Li

**Affiliations:** The Institute of Seawater Desalination and Multipurpose Utilization, MNR, Tianjin 300192, China

**Keywords:** auto-adjusting ejector, MED-TVC, CFD model, retractable needle, solar energy

## Abstract

Ejectors have been widely used in multi-effect distillation, thermal vapor compression (MED-TVC) desalination systems due to their simple structures and low energy consumption. However, traditional fixed geometry ejectors fail to operate under unstable working conditions driven by solar energy. Herein, a dynamic auto-adjusting ejector, equipped with a needle at the nozzle throat, is proposed to improve the ejector’s performance under changeable operating conditions. A two-dimensional computational fluid dynamics (CFD) model is built to analyze the performance and flow field of the ejector. It is found that the achievable entrainment ratio gradually increases as the needle approaches the nozzle, and the entrainment ratio of the ejector is relatively stable, varying slightly between 1.1–1.2 when the primary pressure changes from 2.5 to 4 bar. Besides, the performance comparison between the proposed ejector and the traditional ejector is studied under the same primary pressure range. The entrainment ratio of the designed ejector was 1.6 times higher than that of the conventional ejector at a primary pressure of 2.5 bar. Furthermore, the average entrainment ratio of the designed ejector is 1.14, as compared to 0.84 for the traditional ejector. Overall, the proposed auto-adjusting ejector could be potentially used in MED-TVC desalination systems under variable conditions.

## 1. Introduction

The fast-growing development of seawater desalination and solar energy utilization technology advances the applications of solar seawater desalination technology [1,2,3]. Since the solar concentrated photothermal system has high solar thermal efficiency and can produce a high-grade heat source, the multi-effect distillation (MED) desalination system driven by concentrating photothermal energy has become a focus of research in the field of solar seawater desalination technology [4,5]. Ejectors have been widely used in MED desalination systems due to their simple mechanical structures and the ability to reduce energy consumption [6,7,8]. However, due to the unstable working condition of the concentrated photothermal MED desalination system and its special working situations, most of the ejectors fail to meet the needs of the system. Therefore, it is necessary to develop high-performance ejectors that are suitable for the concentrated photothermal MED desalination system.

Based on the location of the primary nozzle, the ejector can be directly divided into two main types: Constant area mixing (CAM) ejector and constant pressure mixing (CPM) ejector. The CPM ejectors are considered to be more favorable in practical use because of their stable performance at a wider range of back pressures [9]. Therefore, the constant pressure mixing model has always been applied in the ejector design [10]. Keenan et al. [11] employed thermodynamic formulas to derive the calculation model of ejectors for the first time. Additionally, the isobaric mixing theory was widely recognized and applied afterwards. Munday et al. [12] developed this model based on the assumption that the flow mixing progress began at a “fictive throat”, which is located downstream of the primary nozzle exit. Huang et al. [13] modified this model to explain the double choking phenomenon. It was assumed that the “fictive throat” was the location at the constant area section and the mixing progress occurred when the entrained flow was choked. To predict the performance of ejectors more accurately, Zhu et al. [14] developed a “shock circle model” by considering the actual non-uniform velocity distribution of the secondary flow in the mixing chamber. In these studies, limited work has been reported on the effects of the ejector geometry on its performance. The ejector with a designed geometry is desired for fitting the operational conditions. Furthermore, to gain deep insight into the ejector mechanism, many attempts have been made to study the effect of the nozzle exit position (NXP) and area ratio (AR) on the performance of the ejector. Researchers have found that some other geometric shapes of the nozzle, such as mixing section size, diffuser size, diverging angle, and constant area section size, are among the factors that affect the ejector performance [9].

In recent years, great efforts have been made to explore the geometry of ejectors for practical use. Park et al. [15] presented a robust TVC for a pilot-scale desalination system based on the CFD method. It was a novel robust ejector designed to be operated in the critical mode under various operating pressures by changing the primary nozzle throat diameter and the ejector throat diameter. Chaiwongsa et al. [16] investigated the influence of the nozzle throat area on the performance of the ejector and refrigeration system through an experimental study. It was found that the primary mass flow rate varied directly with the nozzle throat, and the secondary mass flow rate also tended to vary directly with the nozzle throat. Zhang et al. [17] investigated the effect of the nozzle position on steam ejectors by placing a long conical regulating cone at the nozzle throat. It was found that the performance of the ejector was adjustable by moving the position of the regulating cone to change the area of the nozzle throat. Liu et al. [18] designed an axial-symmetric CFD model to investigate the effect of the area ratio on ejector performance. Simulation results showed that the entrainment ratio increased by about 20% by adjusting the area ratio from 18.23 to 30.25 as the effective cross-area increased with the throat diameter. Thongtip et al. [19] tested four different nozzles of the same area ratio and two nozzles of different area ratios at various operating conditions. Their research showed that using larger nozzles with a lower generator temperature was beneficial to the ejector performance used in the R141b ejector refrigerator. Wang et al. [20] proposed the concept of the adaptive nozzle exit position (ANXP) ejector. The results indicated that the new method could improve the ejector performance in MED-TVC desalination systems. Tang et al. [21,22,23] carried out a CFD study to optimize the entrainment passage for performance improvement in the MED-TVC desalination system under both design and off-design conditions. Their results showed that the throat-entraining entrance downstream pressure regulation could be adopted if the ejector operates under the design condition; otherwise, the combined entraining entrance downstream pressure regulation would be the best choice for the off-design conditions. According to the effects of both AR and NXP on the performance of the ejector, Wang et al. [24] explored the AR auto-adjusting ejector and the NXP auto-adjusting ejector. The CFD simulations showed that two kinds of auto-tuning ejectors exhibited better performance than that of the constant structure. Pei et al. [25] investigated a wide-operating-range ejector by using hydrogen gas as a working fluid. The experimental data showed that the optimal value range for the diameter ratio was 3.00–3.54 and the lower diameter ratio in optimal value benefited the operating range extension. Gu et al. [26] proposed an auto-tuning AR ejector by installing a spindle in the primary nozzle. Compared with the average entrainment ratio of 0.69 for a conventional fixed geometry ejector, the new design had an average entrainment ratio of 1.39. Wang et al. [27] explored a new design method for the primary nozzle to simplify the design process and improve the overall ejector efficiency. The proposed design method could improve the overall efficiency of the ejector by 14.41% compared to conventional methods. Gao et al. [28] studied the steam ejector used in a MED-TVC desalination system. The CFD simulation showed that adding an auxiliary entrainment inlet at the low-pressure region of the ejector was beneficial to improve its entrainment ratio and outlet mass flow rate. Xue et al. [29] investigated a two-stage vacuum ejector for the MED-TVC desalination system. The experimental results indicated the two-stage ejector could be used at a lower vacuum degree than the single ejector.

Despite the good performance of ejectors in the studies, most of the research on the performance of ejectors has assumed that the ejector works under the designed condition. However, due to the instability of solar energy, it is difficult to ensure that the concentrated photothermal MED desalination systems operate under fixed conditions. Inevitably, the departure from the design condition will result in the deterioration of the ejector performance. While researchers have investigated some structured ejectors that can meet the requirements of off-design conditions to a certain extent, the operating conditions are still limited and the ejector performance is not suited for concentrated photothermal MED desalination. Thus, it is challenging to adapt to the operating environment and the situation where the steam parameters fluctuate greatly.

This work aims to demonstrate a wide-operating-range ejector for the concentrated photothermal MED desalination systems both theoretically and experimentally. Firstly, the thermodynamic properties of the system are obtained by establishing the mathematical model of the dynamic auto-adjusting steam ejector. Then, the operation parameters are used to provide the basis for the structural design of the key components of the dynamic auto-adjusting steam ejector. According to the determined parameters of the whole structure of the steam ejector, an optimized design scheme is generated. Finally, by using the optimal design results of the steam ejector, a dynamic auto-adjusting steam ejector demonstration prototype is developed to elucidate the feasibility of the design scheme.

## 2. Ejector Design

### 2.1. Working Principle of the Traditional Ejector

**Figure 1:**
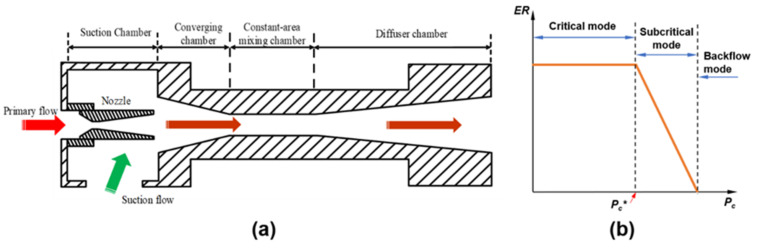
(**a**) The cross-section view of the structure of the ejector; (**b**) the performance curves of the ejector.

The ejector was mainly comprised of a primary flow nozzle, a suction chamber, a converging chamber, a constant-area mixing chamber, and a diffuser chamber (Figure 1a). The high-pressure primary flow is accelerated to supersonic speed when passing through the nozzle. The high-speed primary flow will form a low-pressure area in the suction chamber, and then the secondary flow is sucked into the suction chamber due to the pressure difference. Under the action of the viscous sheer force of the boundary layer, the two fluids are fully mixed through a complex energy and momentum exchange process, and a diagonal shock wave train is generated inside the ejector. At the converging chamber, the working fluid is evenly mixed, the fluid velocity drops rapidly and the pressure rises rapidly to form a positive shock wave in the constant-area mixing chamber. Finally, the fluid goes through the diffusion process in the diffusion chamber and is discharged at the outlet.

The working mode of the ejector can be divided into three types: Critical mode, subcritical mode, and backflow mode, as shown in Figure 1b. When the outlet pressure is lower than the critical back pressure (Pc*), the ejector works efficiently in the critical mode, and the entrainment ratio remains at a constant maximum value. When the outlet pressure is slightly higher than Pc*, the ejector works in a subcritical state, and the entrainment ratio drops rapidly. When the outlet pressure continues to increase, the ejector works in the backflow mode, and the fluid flows back to the suction chamber and cannot work normally. Therefore, the ejector should be designed to work in critical mode to maintain the best entrainment performance.

### 2.2. Design of the Dynamic Auto-Adjusting Ejector

**Figure 2:**
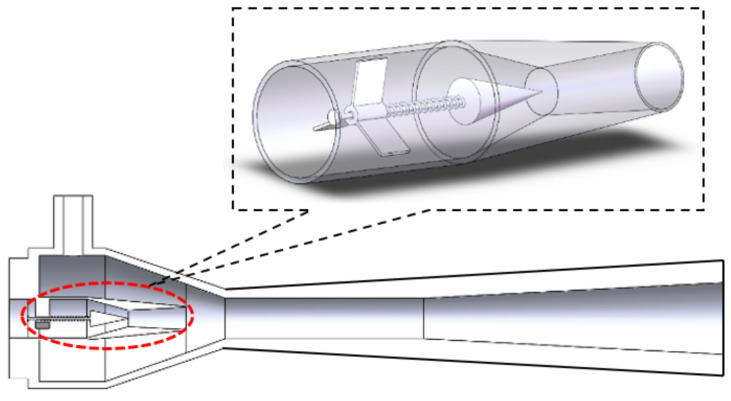
Schematic illustration of the structure of the proposed auto-adjusting ejector. Inset: The magnified illustration of the structure of the nozzle of the ejector.

To meet the requirements of reliable performance of the ejector under various working conditions of primary flow pressure, a dynamic auto-adjusting ejector is designed for variable solar energy-driven conditions. The overall structure of the ejector is shown in Figure 2. Compared to traditional ejectors, the dynamic auto-adjusting ejector is equipped with a retractable needle at the nozzle entrance. The needle and its connecting rod are fixed by a movable shaft and a sleeve structure, and the front and back movement is controlled by a spring. The sleeve is fixed by three support plates connected to the inner wall of the ejector. The high-strength lightweight titanium alloy is used for spray needles and connecting rods to ensure that they will not sag or swing up and down.

The internal structure of the nozzle is displayed in Figure 2 Inset. When the primary pressure increases, the back section of the needle is pushed by the primary high-pressure fluid, and the spring undergoes axial deformation to move the needle toward the nozzle throat leading to a decrease in the nozzle throat area. When the inlet pressure of the ejector decreases, the force of the spring decreases and the needle moves back and the area of the nozzle throat increases. According to this principle, the *AR* of the ejector is expressed as the ratio of the cross-sectional area of the mixing chamber (*A_T_*) and the difference between the cross-sectional area of the nozzle throat (*A_N_*) and the plugging area of the retractable needle (*A_S_*), as given in the following equation:

(1) $$AR=\frac{A_{T}}{A_{N}-A_{S}}$$

The forward and backward movements of the needle are indicated by Δ*L*. When the tip of the needle is at the nozzle throat, Δ*L* is defined as zero, and the right moving is defined as positive. The needle moves 1 mm to the right, the value of Δ*L* is 1 mm, and so on. The structure parameters of the ejector are given in Table 1. The internal angle of the needle is set to 9.5° and Δ*L* is set to zero at the initial position.

The parameters of the entrainment ratio and the compression ratio are used to describe the performance of the ejectors, as provided below. The entrainment ratio (*ER*) is expressed as the ratio between the secondary flow mass flow rate (${\dot{m}}_{s}$) and the primary flow mass flow rate (${\dot{m}}_{p}$), as shown in Equation (2). The value of *ER* in this research is in the range of 0.6–1.2.

(2) $$ER=\frac{{\dot{m}}_{s}}{{\dot{m}}_{p}}$$

The compression ratio (*CR*) is expressed as the static pressure at the exit of the diffuser (*P_c_*) divided by the static pressure of the secondary flow (*P_s_* as given in Equation (3)). The value of *CR* in this work is approximately in the range of 2–2.5.

(3) $$CR=\frac{P_{c}}{P_{s}}$$

## 3. Dynamic Model Analysis of Auto-Adjusting Ejector

### 3.1. Meshing of the Auto-Adjusting Ejector

In this work, the two-dimensional axisymmetric model was employed for the ejector calculation. Sharifi et al. [30] established a calculation model for the traditional steam thermal compression ejector in the seawater desalination system based on two-dimensional axisymmetric and three-dimensional methods, respectively. The simulation results obtained by both models turned out to be similar. Therefore, the application of a two-dimensional axisymmetric model would be sufficient for calculation with the desired accuracy.

**Figure 3:**
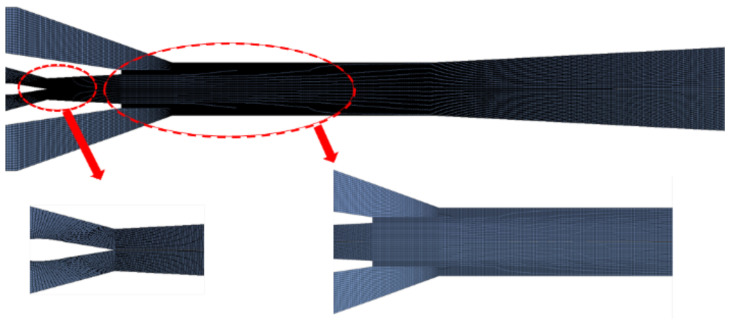
Calculation domain and the meshes of the ejector.

Figure 3 illustrates a schematic diagram of the designed auto-adjusting ejector computational domain grid. To obtain more accurate results, the different region of the ejector model was divided in mesh. At the connection of each region, a same mesh surface was maintained. Additionally, in the key regions near the wall of the ejector, where the fluid state altered greatly with a relatively high velocity gradient, the mesh density was increased accordingly. After verification of grid independence and taking the consumption of computing resources and computing time into consideration, the calculation could be acceptable to meet the accuracy requirement for reflecting the internal flow field of the ejector. The number of grids in the final model is estimated to be 40,000.

### 3.2. Numerical Calculation Model

To simplify the solution process, this article makes the following assumptions:

- The fluid is a steady ideal gas in the ejector;
- The inner wall of the ejector is adiabatic;
- The vortex in the flow field inside the ejector is an isentropic process;
- The primary steam of the ejector is superheated;
- The mixing process of the fluid is completed in the mixing chamber.

Based on the above assumptions, the governing equations, including the mass conservation, energy conservation, and momentum conservation equations, are given as follows:

The conservation of mass equation:

(4) $$A\frac{\partial \rho }{\partial t}+\frac{\partial }{\partial x_{i}}\left(\rho u_{i}A\right)=0$$

The momentum conservation equation:

(5) $$\frac{\partial }{\partial t}\left(\rho u_{i}\right)+\frac{\partial }{\partial x_{j}}\left(\rho u_{i}u_{j}\right)=-\frac{\partial P}{\partial x_{i}}+\frac{\partial \tau_{ij}}{\partial x_{j}}$$

The conservation of energy equation:

(6) $$\frac{\partial }{\partial t}\left(\rho E\right)+\frac{\partial }{\partial x_{i}}\left(u_{i}\left(\rho E+P\right)\right)=\frac{\partial }{\partial x_{i}}\left(\alpha_{eff}\frac{\partial T}{\partial x_{i}}\right)+\frac{\partial }{\partial x_{i}}\left(u_{j}\left(\tau_{ij}\right)\right)$$

In Equations (4)–(6), *i*, *j* = x, y, z was defined according to the Einstein summation rule. The $\tau_{ij}$ and ρ are expressed as follows:

(7) $$\tau_{ij}=\mu_{eff}\left(\frac{\partial u_{i}}{\partial x_{j}}+\frac{\partial u_{j}}{\partial x_{i}}\right)-\frac{2}{3}\mu_{eff}\frac{\partial u_{k}}{\partial x_{k}}\delta_{ij}$$

(8) $$\rho =\frac{P}{RT}$$

where *A* is the cross-sectional area, *ρ* is the density, *T* is the static temperature, *E* is the total energy, *P* is the pressure, *α_eff_* is the thermal conductivity coefficient, *µ_eff_* is the dynamic viscosity coefficient, and *R* is the ideal gas constant, respectively. 

### 3.3. Numerical Settings and Boundary Conditions

The choice of turbulence model is related to the accuracy of the simulation results in the CFD simulations. The *k-ε* model is the most widely applied in CFD research. The *k-ε* models in FLUENT include three types: Standard *k-ε*, RNG *k-ε*, and Realizable *k-ε* [31]. The Realizable *k-ε* model, as an improved version of the standard turbulence model, is better than the traditional Standard *k-ε* model in calculating different fluid flow processes, such as ejection, fluid mixing layer, pipe flow, and boundary layer flow. In particular, the simulation analysis of this model in terms of fluid diffusion velocity is very accurate for the calculation results of the axisymmetric jet model and the results of the plane jet. In addition, the effectiveness of the “Realizable *k-ε*” turbulence model in predicting steam ejectors in seawater desalination systems has been verified by experimental data. Thus, this research uses the “Realizable *k-ε*” turbulence model for the simulation calculation of turbulence in the ejector internal flow field.

The transfer equations of the “Realizable *k-ε*” turbulence model are expressed as follows:

For turbulent kinetic energy *k*:

(9) $$\frac{\partial }{\partial t}\left(\rho k\right)+\frac{\partial }{\partial x_{i}}\left(\rho ku_{i}\right)=\frac{\partial }{\partial x_{j}}\left[\left(\mu +\frac{\mu_{t}}{\sigma_{k}}\right)\frac{\partial k}{\partial x_{j}}\right]+G_{k}+G_{b}-\rho \varepsilon -Y_{M}+S_{k}$$

For turbulent dissipation rate *ε*:

(10) $$\frac{\partial }{\partial t}\left(\rho \varepsilon \right)+\frac{\partial }{\partial x_{i}}\left(\rho \varepsilon u_{j}\right)=\frac{\partial }{\partial x_{j}}\left[\left(\mu +\frac{\mu_{t}}{\sigma_{\varepsilon }}\right)\frac{\partial \varepsilon }{\partial x_{j}}\right]+\rho C_{1}S_{\varepsilon }-\rho C_{2}\frac{\varepsilon^{2}}{k+\sqrt{v\varepsilon }}+C_{1\varepsilon }\frac{\varepsilon }{k}C_{3\varepsilon }G_{b}+S_{\varepsilon }$$

In Equations (9) and (10), *G_k_* is the turbulent kinetic energy produced by the average velocity gradient, *G_b_* is the turbulent kinetic energy produced by rising buoyancy, *µ* is the speed, $Y_{M}$ is the fluctuating expansion parameter incompressible turbulence, *σ_k_* and *σ_ε_* are the turbulent Prandtl numbers of *k* and *ε*, respectively, *C_1ε_* and *C_2_* are fixed constants, and *S_k_* and *S_ε_* are defined constants.

Among which:

(11) $$C_{1}=\max\left[0.43,\frac{\eta }{\eta +5}\right],\eta =S\frac{k}{\varepsilon },S=\sqrt{2S_{ij}S_{ij}}$$

In the turbulence model, the turbulent viscosity ($\mu_{t}$) is calculated by the following formula:

(12) $$\mu_{t}=\rho C_{\mu }\frac{k^{2}}{\varepsilon }$$

To ensure that the model achieves a good calculation, the model constants are selected as follows:

(13) $$C_{\mu }=0.09,C_{1\varepsilon }=1.44,C_{2}=1.90,\sigma_{k}=1.00,\sigma_{\varepsilon }=1.20$$

In this paper, the Standard-Wall-Function is used to solve the near-wall function. The near-wall function is a series of semi-empirical formulas used to calculate the viscous influence area between the wall and the fully developed turbulence in the turbulence problem. Standard-Wall-Function is based on the research of Launder and Spalding and is widely used in fluid simulation in the industry. This function is also suitable for supersonic fluid calculation and has been verified by experimental data.

In the Fluent software, the parameter settings of the fluid model solver inside the ejector are listed in Table 2.

The proposed ejector consists of three fluid inlets and one outlet. The boundary types of the two primary inlet flows and one secondary inlet flow are set as “pressure inlet”, and the ejector outlet is set as the “pressure outlet”. The boundary conditions applied to the inlet are axisymmetric with swirl. The specific parameters of the boundary are summarized in Table 3.

In the process of solving the control equations and turbulence model, the second-order upwind is used to discretize the convection terms, and the SIMPLEC algorithm is used to calculate the pressure field. In the CFD simulation of the ejector, when the residuals of all equations are less than 10^−7^ and the calculated mass flow meets the mass conservation equation (i.e., the obtained net flow is less than 10^−7^ kg/s), the calculation is considered to be converged and ends.

### 3.4. Grid Independence Analysis

**Figure 4:**
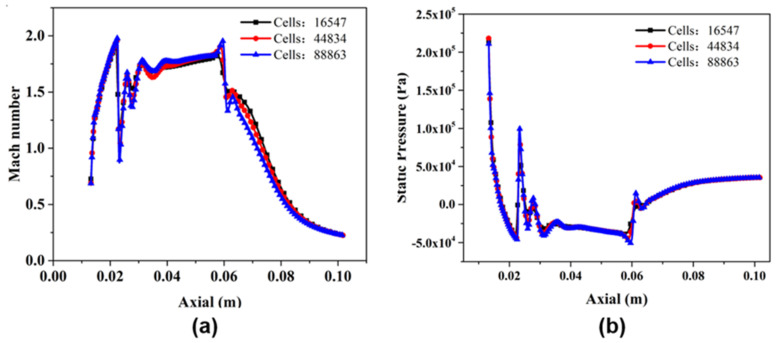
(**a**) Distributions of Mach number along the axial of the ejector; (**b**) distributions of static pressure along the axial of the ejector.

The CFD ejector model needs to be verified for grid independence so that the simulation results are not affected by the number of grids. This work draws three sets of grids, including the grid number of 16547, 44834, and 88863, respectively. Generally, the Mach number and pressure distribution of the fluid inside the ejector determine most of its performance indicators. Therefore, the axial Mach number and axial static pressure are used for the verification of grid independence. In Figure 4a,b, the axial Mach number and axial pressure distribution of the ejector inner fluid are presented, respectively. The Mach number and pressure distribution of the three models with different mesh numbers are basically the same. Only the Mach numbers with x from 0.06 to 0.08 m in Figure 4a have some slight discrepancy. Based on this, the influence of three different mesh number models on the ejection ratio of the ejector is carried out, and the relative error is less than 1.3%. Besides, an excessive number of model grids will consume CPU resources and time to converge in the calculation results. Therefore, in this work, the number of ejector model grids of 44834 was selected, based on the results obtained from the grid independence verification and considering the calculation resources and calculation accuracy of the simulation.

### 3.5. Experimental Platform and Conditions

**Figure 5:**
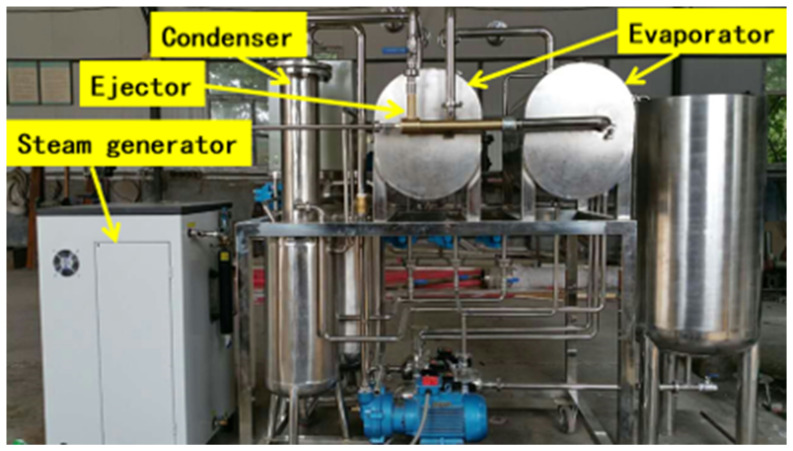
The photograph of the experimental apparatus.

To realize the model verification, a set of the experimental platform was designed and built according to actual application scenarios, combined with the ejector operating conditions, as depicted in Figure 5. The experimental platform was comprised of an ejector, a condenser, a set of evaporators, sensors, data acquisition devices and control systems. During the experimental processes, feed water, which sprays in every evaporator, turned into water vapor and then condensed at low pressure. The latent heat released from the condensation of the vapor is used as a heat source to warm up the brine water. The ejector plays an important role in recycling the vapor from the end to the first effect evaporator. The high temperature and pressure steam were controlled by a valve where the opening can be adjusted manually. The experiment was conducted with a gradual change in the primary flow pressure. The experimental process was relatively simple. Firstly, the air tightness of the devices was checked carefully. Then, the inlet valve of the primary flow was opened gradually, allowing the pressure to fluctuate within the range of 2–4 bar. The pressure was a little higher than the simulation value to offset the loss around the ejector interface. The ejector back pressure and secondary pressure were fixed separately as the experimental comparison. The suction pressure and mass flow rate of all the flows were recorded. Since it is a valid experimental platform for ejectors, the steam source is replaced by an electric steam boiler, which can generate high-pressure steam up to about 10 bar. The steam mass flow at the three ports of the ejector is measured by mass flow meters, and the pressure and temperature are measured by a high-precision pressure–temperature sensor. The pilot experiment system adopts a set of DAQ (Data Acquisition) equipment for data collection, recording, and display.

## 4. Results and Discussion

**Figure 6:**
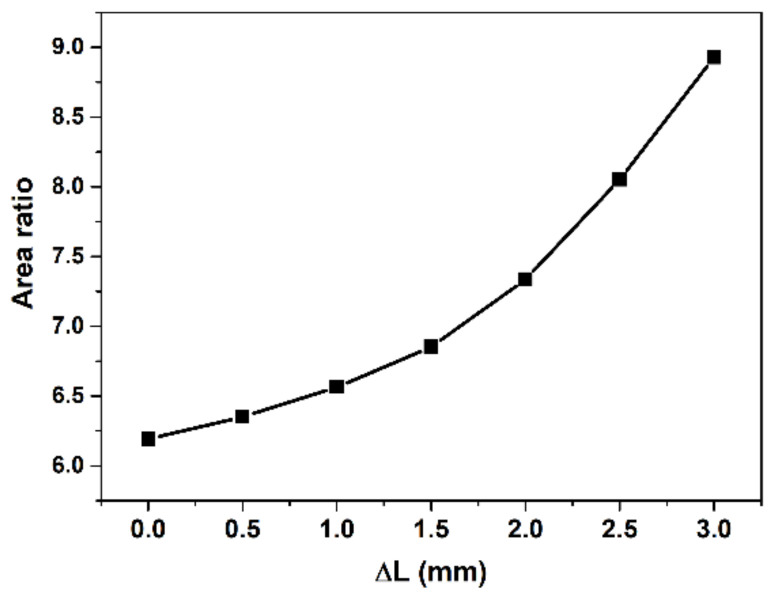
The relation between *AR* and spindle tip position.

To study the performance of the auto-adjusting ejector under variable operating conditions, the CFD simulation method is adopted. The operating conditions and performance parameters of the ejector to be investigated are compression ratio, primary pressure, back pressure, ejection ratio, and *AR*. The correlation curve between the *AR* of the adjustable ejector and the back-and-forth movement of the spray needle is shown in Figure 6.

The relation between *AR* and spindle tip position was fit into a function of:

(14) $$AR=0.3092\Delta L^{2}-0.0433\Delta L+6.2442$$

The expression curve was close enough to calculate the value with the R^2^ of 0.9978. According to the spring elasticity coefficient and Hooke law, the force and the deformation value (elongation or compression) of the spring are proportional within the elastic limit and the formula is defined as:

(15) $$P_{p}=k_{E}\cdot \Delta L$$

The relationship between spring elasticity coefficient ($k_{E}$) and the inlet steam pressure ($P_{p}$) of the ejector and *AR* can be obtained, and the basis for the selection of the spring elasticity coefficient can be estimated by the following formula.

(16) $$AR=0.3092{\left(\frac{P_{p}}{k_{E}}\right)}^{2}-0.0433\frac{P_{p}}{k_{E}}+6.2442$$

### 4.1. Relationship between Needle Position and Ejector Performance

#### 4.1.1. Influence of Needle Position on Ejector Performance

**Figure 7:**
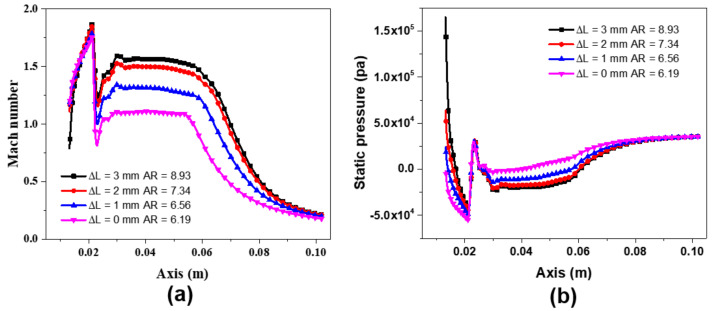
(**a**) Mach number along the ejector axis under different Δ*L*; (**b**) static pressure along the ejector axis under different Δ*L*.

Figure 7a,b showed the distributions of Mach number and static pressure along the ejector axis when the tip position of the needle Δ*L* is 0 mm, 1 mm, 2 mm, and 3 mm, respectively. The boundary conditions are set as primary flow pressure of 3 bar, secondary flow pressure of 0.1 bar, and back pressure of 0.35 bar, respectively. Similar to the traditional ejector, the velocity of the primary fluid increases and reaches the supersonic at the throat of the nozzle and then continues to accelerate and reach the maximum velocity of 1.9 Mach at the exit of the nozzle. In Figure 7a, as the tip position moves, the value of *AR* increases and the Mach number at the nozzle outlet increases, thereby improving the ejection ability to the secondary flow. Meanwhile, the same results can be observed from Figure 7b. With the movement of the needle, the lower the pressure reaches the nozzle outlet, the stronger the suction capacity of the ejector and the lower the velocity of the fluid in the ejector. 

**Figure 8:**
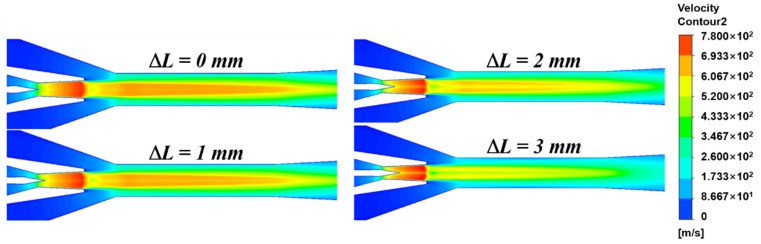
Velocity contours of the ejector under different Δ*L*.

The velocity contours of the internal flow field of the ejector are shown in Figure 8. It is observed that the fluid velocity gradually decreases as the Δ*L* increases. Compared with the single supersonic nucleus of the traditional ejector, the fluid supersonic nucleus of the auto-adjusting ejector is gradually divided into a shape with a lower center velocity and a higher peripheral velocity in the cross-section due to the continuous movement of the needle. Besides, there exists a small area of low velocity at the nozzle exit.

#### 4.1.2. The Influence of Needle Position on Port Flow

**Figure 9:**
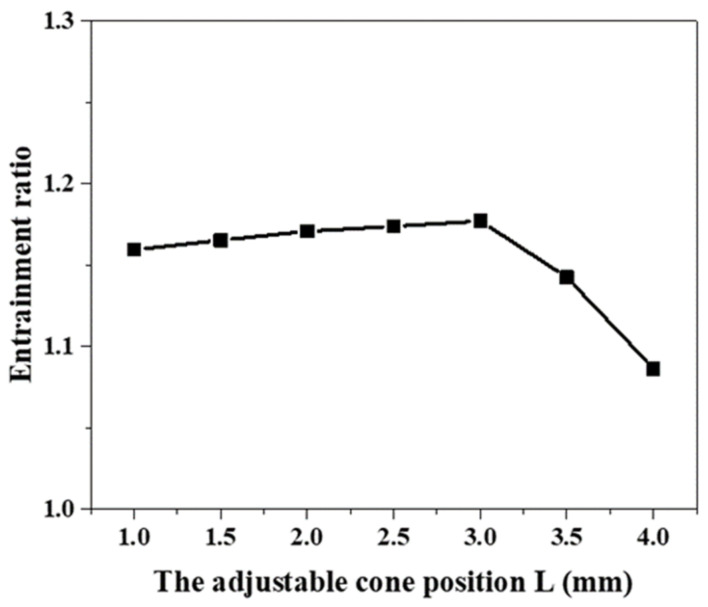
The relationship between the needle position and the entrainment ratio.

The position of the needle of the auto-adjusting ejector move with the fluctuation of the primary flow pressure and the *AR* of the ejector also changes accordingly. The relationship between the position of the needle and the ejector performance is shown in Figure 9. The boundary conditions are set as primary pressure of three bar, secondary pressure of 0.1 bar, and back pressure of 0.35 bar, respectively. It is observed that the performance of the auto-adjusting ejector first increases and then decreases in the fixed working conditions, as the needle continues to move to the throat of the ejector.

**Figure 10:**
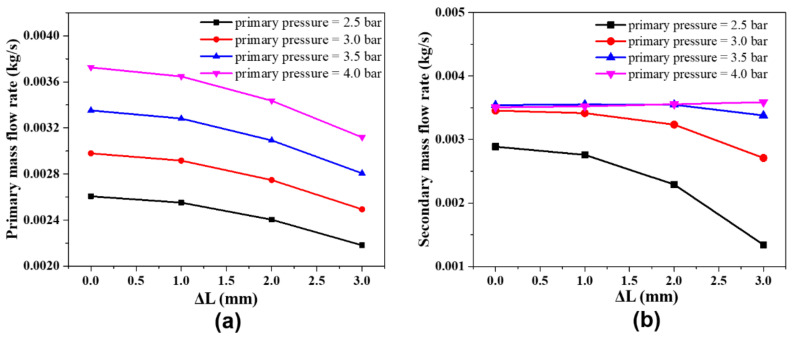
(**a**) The relationship of the primary mass flow rate and needle position; (**b**) the relationship between the secondary mass flow rate and needle position.

Figure 10 shows the diagrams of the relationships between the mass flow of the primary and secondary of the ejector and the needle position under the same operating conditions. It is observed that the mass flow of the primary inlet of the ejector gradually decreases as the tip position of the needle increases. This is because the *AR* of the ejector decreases as the needle continuously deepens leading to the effective throat area of the nozzle decreasing. It shows that the secondary flow mass flow rate gradually decreases and the suction capacity decreases with the needle position continuously increasing as the primary pressure below three bar. Besides, the secondary flow rate of the ejector no longer decreases with the needle position increasing as the primary pressure exceeds 3.5 bar.

### 4.2. Influence of Operating Parameters on Ejector Performance

#### 4.2.1. Influence of Compression Ratio on Ejector Performance

**Figure 11:**
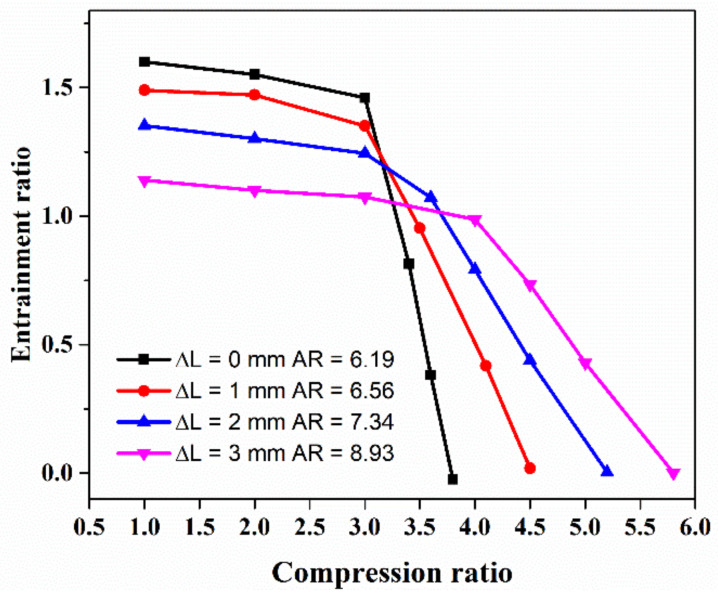
The effect of compression ratio on *ER* with different spindle tip positions.

Figure 11 shows the relationship between the entrainment ratio and the compression ratio of the auto-adjusting ejector when the primary pressure is 2.5 bar under the same operating conditions. It is indicated that the ejector can form effective ejection within the compression ratio range of 0.8–6 bar, and the ejection coefficient of the ejector can reach about 1.5. With the compression ratio ranging from 0.8 to 3 bar, the compression ratio increase has little effect on the ejection efficiency. When the primary flow pressure of the ejector is insufficient or the expansion ratio is too small, the overall ejection coefficient will rapidly decrease, and the ejector will enter a backflow failure state when the compression ratio is greater than 3.5. This phenomenon is similar to the traditional ejector performance curve. When the needle position of the ejector is at 0 mm, a larger compression ratio range is obtained and the maximum entrainment ratio is 1.06 and the maximum compression ratio is 5.5 where the ejector still has the suction ability. As the nozzle moves further, the ejection performance gradually increases, and the effective compression ratio continues to shrink.

#### 4.2.2. Influence of Primary Pressure Change on the Performance

**Figure 12:**
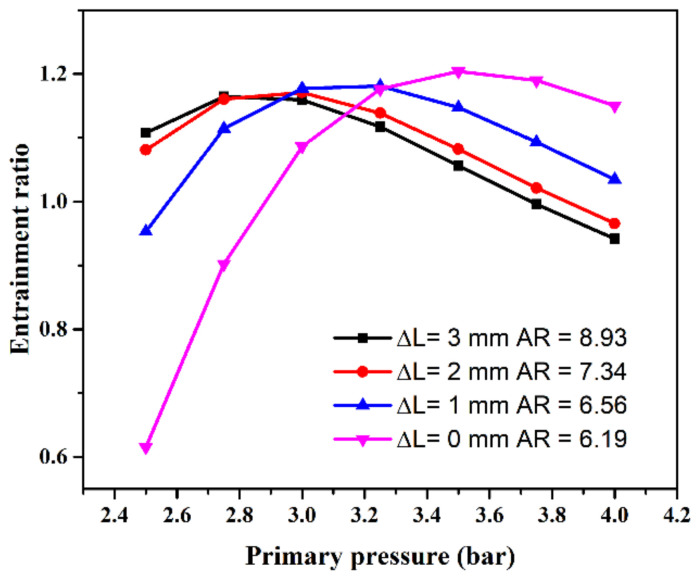
Effect of expansion ratio variation on the entrainment ratio.

Figure 12 shows the effect of expansion ratio variation on the entrainment ratio. The primary pressure changes gradually from 2.5 to 4 bar when the other boundary conditions are set as a secondary pressure of 0.1 bar, the back pressure of 0.35 bar, respectively. It can be seen that the ejector’s primary flow pressure has a huge impact on the ejector’s performance. The better performance of the ejector can be achieved with a smaller value of Δ*L* when the primary flow pressure is less than three bar. The performance of the ejector with the large Δ*L* is gradually improved with the primary pressure increasing. This phenomenon is consistent with the actual operating conditions of the ejector. When the pressure of the primary flow increases, the fluid exerts a thrust force on the spring to the right and pushes the ejector needle to the right and then the Δ*L* increases. During this process, the ejector performance can always be maintained at a high level. When the primary pressure of the auto-adjusting ejector changes, its entrainment ratio increases first and then decreases and changes. The larger the distance is, the better the entrainment ratio obtained.

### 4.3. Performance Comparison with Traditional Ejector

**Figure 13:**
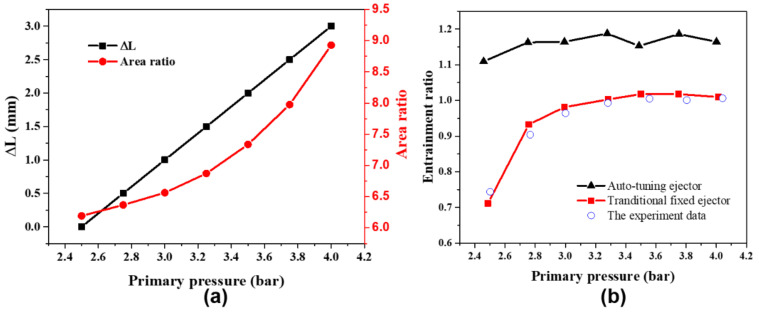
(**a**) The relationship between the primary pressure, Δ*L* and *AR*; (**b**) comparison of *ER* between auto-tuning *AR* ejector and traditional ejector.

The auto-adjusting ejector is compared to the traditional ejector in the primary pressure range of the variable operating conditions. To simplify the analysis, this work assumes that the needle position increases with the primary pressure, as shown in Figure 13a. The two ejectors are set as the same boundary conditions (back pressure 0.35 bar, secondary pressure 0.1 bar, the primary pressure range of 2.5 bar to 4 bar) and the comparison result is shown in Figure 13b. It is observed that the *ER* of the auto-adjusting ejector only changes in a small range between 1.1 and 1.2, but the *ER* of the traditional ejector decreases from 1 to 0.7 in the low-pressure range. The main reason is that the retracting needle allows the ejector to maintain a relatively stable operating state when the *AR* increases with the primary pressure decreasing. The average *ER* of the auto-adjusting ejector can reach 1.14 compared to 0.84 for the traditional ejector. Besides, when the primary flow pressure is 2.5 bar, the auto-adjusting ejector can improve the entrainment ratio by 1.6 times compared to the traditional ejector. Therefore, the performance and stability of the auto-adjusting ejector are superior to the traditional ejector under the variable operating conditions of primary flow driven by solar energy.

## 5. Conclusions

In this work, an auto-adjusting ejector with a needle-fixed spring that is sensitive to the primary pressure variation in a solar-powered desalination system is proposed. The area of the ejector nozzle throat is adapted to achieve better performance by the needle moving forward and backward under different solar-powered primary pressure conditions. The main results are as follows.

(1) The parameter *AR* of the ejector is adapted to the pressure fluctuation of the primary flow steam. The *AR* value decreases with the needle moving into the nozzle as the primary pressure increases. On the contrary, the *AR* increase with the needle retracting from the nozzle when the primary pressure decreases. During this process, the ejector can always be maintained in a high-performance operating state.
(2) The average *ER* of the auto-adjusting ejector can reach 1.14 compared with 0.84 for the traditional ejector. The auto-adjusting ejector can improve the entrainment ratio by 1.6 times compared to the traditional ejector. Therefore, the performance and stability of the auto-adjusting ejector are superior to the traditional ejector under the variable operating conditions of the primary flow driven by solar energy.

In this study, a preliminary theoretical and experimental study of the dynamic auto-adjusting ejector was carried out. Additionally, it focuses on the numerical simulation study of the ejector. The elastic modulus and stiffness coefficient of the ejector spring still need further study. The application of the auto-adjusting ejector in the solar-driven low-temperature MED desalination unit will be explored in our future work to investigate the performance of the ejector under the condition of a steam variation, so as to promote the commercial application of this technology.

## Figures and Tables

**Table 1:** Key geometric parameters of the ejector.

Parameters	Value (mm)
Nozzle length	15.71
Nozzle throat diameter	2.42
Nozzle extension diameter	3.31
Suction chamber diameter	18.64
Mixing tube diameter	10.22
Mixing tube length	5.85
Constant-area chamber length	29.38
Constant-area chamber diameter	5.98
Diffuser extension diameter	10.68
Diffuser length	45.00

**Table 2:** Fluent specific parameters set.

Items	Setting
solution method	pressure-based
solver	segregated-implicit
time	steady
velocity formulation	absolute
formulation	implicit
viscous model	realizable *k*-*ε*
near-wall treatment	standard wall function
working medium	water-vapor

**Table 3:** Boundary conditions of the ejector.

Type	Temperature (°C)	Pressure (Bar)
primary flow	133.5	3.0
secondary flow	45.8	0.1
discharge flow	72.7	0.35

## Data Availability

Not applicable.

## References

[1] Zheng Y., Zhao Y., Liang S., Zheng H. (2018). Thermo-Economic Optimization of an Idealized Solar Tower Power Plant Combined with MED System. Entropy.

[2] Wang M., Xu G., An Z., Xu K., Qi C., Das R., Zhao H. (2022). Hierarchically structured bilayer Aerogel-based Salt-resistant solar interfacial evaporator for highly efficient seawater desalination. Sep. Purif. Technol..

[3] Liu H., Huang Z., Liu K., Hu X., Zhou J. (2019). Interfacial Solar-to-Heat Conversion for Desalination. Adv. Energy Mater..

[4] Alhaj M., Tahir F., Al-Ghamdi S.G. (2021). Life-cycle environmental assessment of solar-driven Multi-Effect Desalination (MED) plant. Desalination.

[5] Aly S., Manzoor H., Simson S., Abotaleb A., Lawler J., Mabrouk A.N. (2021). Pilot testing of a novel Multi Effect Distillation (MED) technology for seawater desalination. Desalination.

[6] Al-Mutaz I.S., Wazeer I. (2014). Current status and future directions of MED-TVC desalination technology. Desalination Water Treat..

[7] Ghaffour N., Missimer T.M., Amy G.L. (2013). Technical review and evaluation of the economics of water desalination: Current and future challenges for better water supply sustainability. Desalination.

[8] Samaké O., Galanis N., Sorin M. (2017). Thermo-economic analysis of a multiple-effect desalination system with ejector vapour compression. Energy.

[9] Tashtoush B.M., Al-Nimr M.A., Khasawneh M.A. (2019). A comprehensive review of ejector design, performance, and applications. Appl. Energy.

[10] He S., Li Y., Wang R. (2009). Progress of mathematical modeling on ejectors. Renew. Sustain. Energy Rev..

[11] Keenan J.H., Neumann E.P., Lustwerk F. (1950). An Investigation of Ejector Design by Analysis and Experiment. J. Appl. Mech..

[12] Munday J.T., Bagster D.F. (1977). A New Ejector Theory Applied to Steam Jet Refrigeration. Ind. Eng. Chem. Process Des. Dev..

[13] Huang B.J., Chang J.M., Wang C.P., Petrenko V.A. (1999). A 1-D analysis of ejector performance. Int. J. Refrig..

[14] Zhu Y., Cai W., Wen C., Li Y. (2007). Shock circle model for ejector performance evaluation. Energy Convers. Manag..

[15] Park I. (2009). Robust numerical analysis based design of the thermal vapor compressor shape parameters for multi-effect desalination plants. Desalination.

[16] Chaiwongsa P., Wongwises S. (2007). Effect of throat diameters of the ejector on the performance of the refrigeration cycle using a two-phase ejector as an expansion device. Int. J. Refrig..

[17] Zhang K., Shen S., Yang Y., Tian X. (2012). Experimental Investigation of Adjustable Ejector Performance. J. Energy Eng..

[18] Liu J., Wang L., Jia L., Wang X. (2017). The influence of the area ratio on ejector efficiencies in the MED-TVC desalination system. Desalination.

[19] Thongtip T., Aphornratana S. (2017). An experimental analysis of the impact of primary nozzle geometries on the ejector performance used in R141b ejector refrigerator. Appl. Therm. Eng..

[20] Wang C., Wang L., Wang X., Zhao H. (2017). Design and numerical investigation of an adaptive nozzle exit position ejector in multi-effect distillation desalination system. Energy.

[21] Tang Y., Liu Z., Shi C., Li Y. (2018). A novel steam ejector with pressure regulation to dredge the blocked entrained flow for performance improvement in MED-TVC desalination system. Energy Convers. Manag..

[22] Tang Y., Liu Z., Shi C., Li Y. (2018). A novel steam ejector with pressure regulation to optimize the entrained flow passage for performance improvement in MED-TVC desalination system. Energy.

[23] Tang Y., Liu Z., Li Y., Shi C., Lv C. (2019). A combined pressure regulation technology with multi-optimization of the entrainment passage for performance improvement of the steam ejector in MED-TVC desalination system. Energy.

[24] Wang L., Liu J., Zou T., Du J., Jia F. (2018). Auto-tuning ejector for refrigeration system. Energy.

[25] Pei P., Ren P., Li Y., Wu Z., Chen D., Huang S., Jia X. (2018). Numerical studies on wide-operating-range ejector based on anodic pressure drop characteristics in proton exchange membrane fuel cell system. Appl. Energy.

[26] Gu W., Wang X., Wang L., Yin X., Liu H. (2019). Performance investigation of an auto-tuning area ratio ejector for MED-TVC desalination system. Appl. Therm. Eng..

[27] Wang K., Wang L., Jia L., Cai W., Gao R. (2019). Optimization design of steam ejector primary nozzle for MED-TVC desalination system. Desalination.

[28] Gao S., Zhao H., Wang X., Yu Z., Lai Y. (2019). Study on the performance of a steam ejector with auxiliary entrainment inlet and its application in MED-TVC desalination system. Appl. Therm. Eng..

[29] Xue H., Wang L., Jia L., Xie C., Lv Q. (2019). Design and investigation of a two-stage vacuum ejector for MED-TVC system. Appl. Therm. Eng..

[30] Sharifi N., Boroomand M. (2013). An investigation of thermo-compressor design by analysis and experiment: Part Validation of the numerical method. Energy Convers. Manag..

[31] ANSYS Inc. (2009). ANSYS FLUENT 12.0 Theory Guide. https://usermanual.wiki/Document/ANSYSTheoryGuide.39891911.

